# Impact of mild COVID-19 on balance function in young adults, a prospective observational study

**DOI:** 10.1038/s41598-022-16397-8

**Published:** 2022-07-16

**Authors:** Agnieszka Guzik, Andżelina Wolan-Nieroda, Maciej Kochman, Lidia Perenc, Mariusz Drużbicki

**Affiliations:** Department of Physiotherapy, Institute of Health Sciences, College of Medical Sciences, University of Rzeszów, Rzeszów, Poland

**Keywords:** Neurology, Neurological disorders

## Abstract

Balance is of essential importance in human life. The aim of the study is to examine the incidence of balance impairments in young adults who have recovered from mild COVID-19. The study involved 100 subjects, divided into two groups: the study group (50 individuals) comprised subjects who had recovered from mild COVID-19, and the control group (50 individuals) consisted of healthy subjects matched for gender and age. Balance was assessed using a force platform and clinical tests such as: timed up and go test, 15-s step test, sit-to-stand test and 6-min walk test. The assessment on the platform showed greater balance impairments in the trials with eyes closed; more specifically, compared to the controls, in trials with double-leg support the subjects from the study group acquired significantly higher scores in X average (lateral coordinates) (p < 0.05), Path length, V average (average Centre of Foot Pressure Velocity) (p < 0.05) and Area circular (p < 0.01), with even more significant results in trials with single-leg support in X average (p < 0.001), Y average (anterior–posterior coordinates) (p < 0.001) and Path length (p = 0.004). Higher scores in the timed up and go test were found in the study group (p = 0.013). The control group had higher scores in the remaining tests. The current findings show that mild COVID-19 may lead to balance impairments in young adults. Statistically significant differences in balance were found between the subjects in the study group and the healthy controls. Further studies in this area should take into account more age groups, and patients recovered from severe COVID-19, and should investigate long-term consequences of COVID-19 reflected by balance problems.

## Introduction

With the advancing COVID-19 pandemic, researchers have increasingly focused on nervous system disorders and dysfunctions linked to this condition, due to which a new medical term, Neuro-COVID, has been introduced^1^. Despite the growing body of evidence related to neurological symptoms in patients who have recovered from COVID-19, there are no definitive estimates related to the incidence of nervous system impairments potentially associated with this disease. The related research findings are far from unanimous: the reported incidence rates for any neurological manifestations in patients with COVID-19 range from 36.4^2^ to 84.5%^3^. Furthermore, despite the advancing research related to this disease, and the successive years of the pandemic, we still do not know all of the long-term consequences of SARS-COV-2 infection^4^. It is anticipated that, in the long run, many patients who recovered from COVID-19 may suffer from lasting impairments, including those related to the nervous system^5,6^. Studies also suggest that SARS-CoV-2 infection may affect the autonomic nervous system causing dysautonomia^7–9^ in patients with long COVID syndrome or with post-acute COVID sequalae. It is likely, however, that the autonomic symptoms may also be present in patients recovered from mild COVID-19, and they may well correlate with fatigue or postural/orthostatic intolerance^7–9^.

Postural stability is of essential importance in human life. The central nervous system (CNS), which is responsible for balance integration, relies on information provided by the ocular, vestibular, and proprioceptive systems^10^. Balance disorders increase the risk of falls and may cause serious injuries, such as fractures^11–13^ or brain trauma^14,15^. Falls can be dangerous, as they often lead to disability^16^ or even death, especially in the elderly^13,15^. Given the risk of damage to the CNS due to SARS-CoV-2 infection, it has been suggested that a balance assessment should be performed in COVID-19 survivors, representing different age groups and severity levels of COVID-19^17,18^.

Based on clinical observations and case reports, it has been hypothesised that SARS-CoV-2 infection may affect some of the systems involved in postural control^17–21^. However, balance evaluations in those affected by COVID-19 have mainly been performed using subjective rather than objective methods. To the best of our knowledge, at this point in the world literature there are few studies assessing postural stability in adults and young adults who recovered from COVID-19^17–21^ and there is only one research report, from Istanbul, related to measurement of balance after recovery from COVID-19 performed using objective assessment methods^18^. Furthermore, in Poland no studies related to this issue have been reported so far, consequently it seemed reasonable to undertake research investigating the incidence of this impairment in a group of young adults who have suffered from COVID-19.

A review of the literature suggests that problems associated with COVID-19 attract a lot of attention in our country^22–29^. Polish researchers, however, have mainly focused on such aspects as population policy during the pandemic^22^, social response and spatial mobility change due to pandemic^23^, eating behaviours, level of physical activity, hours of sleep, as well as children’s, adolescents’ and adults’ screen time before and during the pandemic^24–27^, occupational burnout in clinical hospitals during pandemic^28^ or consequences of SARS-CoV-2 fears^29^. On the other hand, no studies have yet investigated the relationship between SARS-CoV-2 infection and such neurological impairments as balance disorder.

The purpose of the study was to assess impairments to postural stability in young adults who had recovered from mild COVID-19.

## Materials and methods

### Study design and participants

This prospective observational study was conducted at the University of Rzeszow, and involved a population living in south-eastern Poland. It assessed 100 individuals (50 females and 50 males) who were divided into two groups depending on whether or not they had been infected with SARS-CoV-2.

The inclusion criteria to the COVID-19 group were as follows: informed consent to participate in the study; confirmed mild infection with SARS-CoV-2 with such symptoms as cough, low-grade fever, taste disturbance, anosmia, or myalgia, but no pulmonary involvement, dyspnoea or cytokine storm requiring hospitalisation or treatment at intensive care units; no steroid or antibiotic therapy received in connection to COVID-19; time from SARS-CoV-2 infection—a minimum of one month; age 19–26 years; no injuries to lower limbs during 12 months preceding enrolment into the study; no orthopaedic disorders affecting the lower limbs (including decreased length of the lower limbs); no diagnosed neurological diseases or disorders (including labyrinth dysfunction); no long-term pharmacotherapy or chronic conditions potentially affecting balance. The exclusion criteria were as follows: lack of informed consent to participate in the study; no confirmed infection with SARS-COV-2; severe case of COVID-19 with pulmonary involvement, dyspnoea or cytokine storm requiring hospitalisation or treatment at an intensive care unit facility; age outside the range of 19 and 26 years; confirmed injuries to lower limbs during 12 months preceding enrolment into the study; as well as confirmed neurological diseases or other chronic conditions as well as pharmacotherapy potentially impairing balance.

The study group comprised 50 subjects who had tested positive and recovered from mild COVID-19, whereas the control group, matched to the study group for gender and age, included 50 healthy subjects who had not suffered from COVID-19. The mean age in the study group was 22.13 years ± 1.53 years, and in the control group 22.27 years ± 0.73 years. No statistically significant differences were found in the distribution of the subjects relative to sex, age and BMI in the two groups. The subjects in the study group were infected on average 4.65 ± 1.6 months earlier, with symptoms of the infection persisting on average for 16.03 ± 9.67 days, and the feeling of fatigue persisting on average for 2.98 ± 2.32 weeks. No participant reported postural orthostatic tachycardia syndrome (POTS), labile blood pressure, heart rate variability dysfunction, impotence or bladder dysfunction. Table 1 presents the characteristics of the groups.

**Table 1:** Baseline characteristics of study and control groups.

	Study group (n = 50)	Control group (n = 50)
Age (years), mean (sd)	22.13 (1.53)	22.27 (0.73)
Sex (female/male)	25/25	25/25
Height (cm), mean (sd)	172.99 (6.60)	173.21 (7.84)
Weight (kg), mean (sd)	70.93 (9.18)	69.36 (10.22)
BMI (kg/m^2^), mean (sd)	23.35 (1.49)	23.10 (1.66)
Time from infection, in months, mean (sd)	4.65 (1.60)	–
Duration of symptoms, in days, mean (sd)	16.03 (9.67)	–
Persistence of fatigue, in weeks, mean (sd)	2.98 (2.32)	–

*sd* standard deviation, *BMI* body mass index.

The study protocol was approved by the local Bioethics Commission at the University of Rzeszow and was registered with ClinicalTrials.gov Registry (Identifier: NCT04934085, 22/06/2021). The study design complied with the Declaration of Helsinki. All the study participants gave their informed consent in writing.

### Procedure

The examination was performed once, at the premises of the University of Rzeszów (in the Biomechanics Laboratory of the Institute of Health Sciences). All the assessments were performed before noon, in uniform conditions and using the same measurement devices. Initially, the participants were evaluated using questionnaires. The first questions were related to personal information, i.e., age, and sex. These were followed with questions related to the time from infection (in months), duration of symptoms (in days), persistence of fatigue (in weeks). Subsequently, the subjects’ height [cm] and weight [kg] were measured. Finally, the participants' balance was assessed using force platform and clinical tests. The measurement of postural stability was followed with clinical tests (detailed description of the postural assessment procedure is presented in “Postural assessment”).

### Postural assessment

Postural stability was assessed with an AccuGait force plate from Advanced Medical Technology Inc. used in combination with AMTI’s Balance Clinic software. Assessment on the platform involved continuous measurement of the Centre of Foot Pressure (COP). By recording body sway deviations, it was possible to acquire accurate information on postural balance. COP movements corresponded to the Centre of Mass movements (COM) in the frontal and sagittal planes. The measures taken into account in the analyses included the Average Load Point X, determining the lateral coordinates X (X average, in cm), the Average Load Point Y determining the anterior–posterior coordinates Y (Y average, in cm), Path Length (cm) of the COP during the trial, Average COP velocity (V average, in cm/s) and Area Circular, i.e., the area defined by the COP during the trial (cm^2^). Stabilography measurements, each continued for 30 s, were carried out during the following trials: with double-leg support and eyes open/closed, and with single-leg support (right/left leg) with eyes open/closed. There was an interval of 30 s between each trial, to avoid fatigue. The assessments were carried out in a closed room, to minimise disturbances or noise. During the assessment, the subjects were asked to stand on the platform and focus their gaze on a red target placed at a distance of 4 ft (1.2 m) on the wall in front. Research has demonstrated that the force plate used in the current study is a valid instrument of a “gold standard” quality^30^.

The clinical tests applied to assess balance included: timed up and go test, 15-s step test, and sit-to-stand test. During the timed up and go test, the subjects are instructed to get up from a chair with a standard-height backrest and to walk 3 m, turn round at a specified location, return to the chair and resume the sitting position unassisted^31^. During the 15-s step test, the subject is required to make as many steps as they can, with the whole foot climbing up and down a 7.5 cm high bench^32^. During the sit-to-stand test, the subject performs the activity of standing up from and sitting down on a 43.2 cm tall chair, as many times as possible in 30 s^33^. Moreover, assessment of walking distance was performed using a 6-min walk test. During the trial, the subjects walked at a self-selected speed for 6 min, between two points located 30 m apart. The distance was measured in metres^34^.

### Statistical analysis

The statistical analyses of the collected material were computed using StatSoft’s Statistica 13.3 package. Distributions of the investigated variables were examined for normality using the Shapiro–Wilk W-test. The Mann–Whitney U-test was used to evaluate the significant differences between the groups. The significance threshold level of p < 0.05 was assumed.

A sample size calculator (“PLUS module” from Statistica 13.3 software) was applied to determine the minimum sample size for the population studied, a sample size of 43 individuals was obtained, 50 individuals were enrolled to the study group.

## Results

### Assessment of balance with the force plate

Assessment of postural stability on the force plate in trials with double-leg support and eyes open showed that the subjects from the study group had significantly higher results in Path length and V average (p < 0.001). No statistically significant differences between the groups were identified in the other measures. In trials with double-leg support and eyes closed, the subjects in the study group achieved significantly higher results than the controls in the measures of X average (p = 0.022), Area circular (p = 0.002), Path length (p = 0.035) and V average (p = 0.026) (Table 2).

**Table 2:** Results of postural stability measurement on the platform with double-leg support in study and control groups.

Variables in postural stability	Study group		Control group		Z	p
	Mean	Sd	Mean	sd		
**Eyes open**						
X average (cm)	−0.81	1.18	−0.52	1.10	−1.36	0.174
Y average (cm)	−3.63	2.48	−2.75	2.07	−1.35	0.178
Area circular (cm^2^)	2.13	1.26	2.27	1.72	0.84	0.402
Path length (cm)	37.96	9.21	32.05	8.27	−4.58	< 0.001
V average (cm/s)	1.27	0.31	1.07	0.28	−4.52	< 0.001
**Eyes closed**						
X average (cm)	−1.01	1.46	−0.08	2.22	−2.28	0.022
Y average (cm)	−3.33	2.46	−2.54	1.73	−1.10	0.272
Area circular (cm^2^)	2.76	1.40	2.18	1.27	−3.12	0.002
Path length (cm)	46.46	16.99	40.52	10.29	40.52	0.035
V average (cm/s)	1.55	0.57	1.35	0.34	−2.23	0.026

*Z *Mann–Whitney U-test result, *sd* standard deviation, *X average *average load point X which determined lateral coordinates X (cm), *Y average *average load point Y which determined the anterior–posterior coordinates Y (cm), *V average *average COP velocity (cm/s), *p *significance level, p < 0.05 reflects statistically significant relationship, p < 0.01 reflects highly significant relationship, p < 0.001 reflects very highly significant relationship.

Measurement of balance in trials with single-leg support (right leg) and eyes open identified no differences between the two groups (p > 0.05). Assessment of balance with single-leg support (left leg) and eyes open showed that the subjects in the study group on average achieved significantly higher results than the controls in the following measures: X average (p = 0.013), Path length (p = 0.002) and V average (p = 0.002) (Table 3).

**Table 3:** Results of postural stability measurement on the platform with single-leg support in study and control groups.

Variables in postural stability	Study group		Control group		Z	p
	Mean	sd	Mean	sd		
**Eyes open (right leg)**						
X average (cm)	6.58	2.13	6.26	3.78	0.15	0.877
Y average (cm)	−1.10	2.55	−1.39	3.08	1.00	0.316
Area circular (cm^2^)	7.74	2.10	8.37	3.54	0.26	0.795
Path length (cm)	116.78	35.78	117.79	31.44	−0.45	0.654
V average (cm/s)	3.89	1.19	3.93	1.05	−0.49	0.624
**Eyes open (left leg)**						
X average (cm)	−7.17	3.03	−5.95	2.76	2.49	0.013
Y average (cm)	−5.92	3.58	−5.23	34.86	−0.32	0.750
Area circular (cm^2^)	9.04	12.57	9.57	9.14	−1.24	0.215
Path length (cm)	119.22	39.66	98.04	29.47	−3.14	0.002
V average (cm/s)	3.97	1.32	3.27	0.98	−3.15	0.002
Eyes closed (right leg)						
X average (cm)	−3.23	3.51	−0.51	2.02	−4.71	< 0.001
Y average (cm)	−4.05	3.13	−2.13	1.67	−3.33	0.001
Area circular (cm^2^)	3.32	3.54	2.28	1.03	0.40	0.692
Path length (cm)	51.59	29.41	42.70	11.31	0.55	0.581
V average (cm/s)	2.06	1.80	1.41	0.37	0.92	0.359
Eyes closed (left leg)						
X average (cm)	−6.29	3.60	−2.94	3.18	−4.82	< 0.001
Y average (cm)	−2.99	4.42	−0.64	3.13	−3.97	< 0.001
Area circular (cm^2^)	8.43	3.72	8.04	6.42	1.28	0.200
Path length (cm)	104.41	31.67	87.34	18.82	2.89	0.004
V average (cm/s)	4.25	1.80	3.64	1.11	1.29	0.199

*Z *Mann–Whitney U-test result, *sd* standard deviation, *X average *average load point X which determined lateral coordinates X (cm), *Y average *average load point Y which determined the anterior–posterior coordinates Y (cm), *V average *average COP velocity (cm/s), *p *significance level, p < 0.05 reflects statistically significant relationship, p < 0.01 reflects highly significant relationship, p < 0.001 reflects very highly significant relationship.

Assessment of postural stability in trials with single-leg support (right leg) and eyes closed showed significantly higher mean results in the study group, compared to the controls, in the measures of X average (p < 0.001) and Y average (p = 0.001). Measurement of balance in trials with single-leg support (left leg) and eyes closed identified significantly higher mean results in the study group, compared to the controls, in the case of X average (p < 0.001), Y average (p < 0.001) and Path length (p = 0.004) (Table 3).

### Assessment of balance using clinical tests

Analysis of the scores acquired by the subjects in the clinical tests showed that there were statistically significant differences in the results between the study group and the controls. The score in the timed up and go test was higher in the study group (p = 0.013). The other scores were higher in the control group, i.e., step test for the left and the right side (p < 0.001), sit-to-stand test (p < 0.001) and 6-min walk test (p = 0.003)—Table 4.

**Table 4:** Results of the clinical tests in study and control group.

	Study group		Control group		Z	p
	Mean	sd	Mean	sd		
Timed Up&Go test (s)	6.19	1.03	5.80	0.87	2.47	0.013
Step test left leg	9.58	3.73	16.29	4.45	−7.18	< 0.001
Step test right leg	9.17	4.08	16.59	5.39	−6.93	< 0.001
Sit-to-stand test	15.32	3.61	19.58	4.73	−5.86	< 0.001
6-min walk test (m)	698.54	98.77	753.31	105.80	2.97	0.003

*Z *Mann–Whitney U-test result, *sd* standard deviation, *p *significance level, p < 0.05 reflects statistically significant relationship, p < 0.01 reflects highly significant relationship, p < 0.001 reflects very highly significant relationship.

## Discussion

The present study shows significant differences in postural stability between 50 individuals recovered from mild COVID-19 and 50 healthy controls, which suggests that COVID-19 can cause balance impairment in young adults. In conducting the assessment, we applied both objective tools (a force platform) and subjective methods (clinical tests). We selected reliable clinical tests, such as the Timed Up and Go Test, which can effectively be used in identifying fall risk in individuals with vestibular dysfunction^31^.

The mean age of the subjects in the study group was 22.13 years ± 1.53 years, and in the control group—22.27 years ± 0.73 years. Being aware that young adults are less at risk of COVID-19 infection, we decided to investigate this age group exclusively in order to acquire clear-cut evidence related to the problem of post-COVID-19 balance impairment which would not be confounded by such factors as co-existing chronic conditions or long-term-medication possibly affecting postural control. Indeed, as anticipated, our findings show balance impairments in young Polish adults who have recovered from mild COVID-19. This may be linked to the involvement of systems responsible for postural stability, as suggested by Yılmaz et al., who investigated a group of subjects on average 10 years older than our sample^18^.

To the best of our knowledge their study was the only one to assess balance in COVID-19 survivors using objective assessment methods. The researchers examined 37 individuals who had recovered from COVID-19 (mean age 32 ± 11 years) and 30 controls who had not experienced the disease (mean age 28 ± 4 years). In addition to the self-report Dizziness Handicap Inventory, the researchers applied three instrumented testing techniques. They reported significantly lower composite and visual general scores in Computerized Dynamic Posturography assessment in the COVID-19 group, compared to the controls (p < 0.01). The Video Head Impulse Test gains identified in the COVID-19 group were considerably reduced in the vertical semi-circular canals, compared to the healthy controls (p < 0.01). The subjects in the COVID-19 group and the control group differed significantly as regards the absence of Vestibular Evoked Myogenic Potentials (p < 0.01). The authors concluded that symptoms attributable to COVID-19 include dizziness but not incapacitating vertigo. This corresponds to the evidence showing that dizziness is reported by one in five adult patients who recovered from COVID-19 but were not hospitalised. This symptom, according to these researchers, may be linked to the involvement of visual and vestibular systems, or their central connections. It is also likely that the changes affecting the systems involved in postural control are irreversible, given the fact that the related symptoms are observed after recovery from COVID-19^18^.

That same study suggests that SARS-CoV-2 infection may, with varied severity, affect the systems involved in postural control, for instance leading to dysfunctions of inner ear organs (utricle or saccule), or to impairments of the vestibular nerve, or extraocular muscles and their central connections^18^. In our study greater impairments in postural stability on the force platform, with both double and single-leg support, were observed in trials with eyes closed, i.e., in trials with double-leg support the subjects in the study group acquired significantly higher results than the controls in X average, Path length, V average (p < 0.05) and Area circular (p < 0.01), and in trials with single-leg support in X average, Y average (p < 0.001) and Path length (p = 0.004). In view of this, it can be assumed that visual control plays an important part while proprioceptive senses may be impaired in patients recovered from mild COVID-19.

Subjects in our study group also reported fatigue, which is a common characteristic in long-COVID, shown by a number of researchers^35–37^. The study by Ferraro et al. provides a detailed description of functional consequences in post-COVID-19 patients, showing that a patient-tailored rehabilitation program, including e.g., balance and coordination exercises, such as one-legged stance, is essential for reducing fatigue and improving functional outcome in activities of daily living (ADL)^35^. Indeed, effective postural control is crucial for proper functioning in ADL. Furthermore, it can be speculated that the balance impairments identified by our study in young adults recovered from mild COVID-19 may be associated with fatigue, also reported by the subjects. This possibly may result from musculoskeletal symptoms which are rather common in patients with COVID-19^37^, in addition to the widely reported neurological symptoms^1–4,38^. Halpin et al. also showed that fatigue was the most common symptom in COVID-19 patients; however, following a rehabilitation program over 70% of the subjects no longer experienced fatigue^39^. Given this, rehabilitation of post-COVID-19 patients is vital for their recovery from fatigue, for improvement in their functional status and consequently, we assume, it may be crucial for improving balance. It is, however, necessary to continue related research, to verify this hypothesis.

In view of the above, it can be expected that rehabilitation programs may also prove effective in improving postural stability of patients recovered from mild COVID-19. Therefore, further research is needed to investigate this specific issue.

The most common symptoms of the disease in the study group included weakness and loss of smell. These problems may be linked to circulatory disturbances, or invasion of the CNS via circulation or the olfactory bulb. During the interview, the subjects reported that weakness adversely affected their functional independence, which is conditioned by lower limb muscle strength^33,40^. Thus, it seemed important to assess the relationship between lower limb strength and COVID-19. In the present study, the relevant measurements were performed using the sit-to-stand test, which enables reliable and easily reproducible assessment of lower limb strength and balance control in both healthy adults and individuals with pathologies^33,40^. Our findings show that subjects who recovered from mild COVID-19 acquired significantly poorer scores in the sit-to-stand test compared to the healthy controls (p < 0.001). Functional independence is also related to walking capacity. We used the 6-min walk test, which is commonly applied in clinical practice to assess exercise capacity in patients with cardiopulmonary or neuromuscular disorders^34^. In this case the scores acquired by the subjects in the study group were also significantly lower (p = 0.003), reflecting their poorer walking capacity.

The current findings show that young adults living in south-eastern Poland present with balance impairment after recovery from mild COVID-19. Our findings suggest there is a need for more in-depth research investigating postural outcomes in COVID-19 survivors. As for the practical implications of this study, the findings show that it is necessary to develop balance rehabilitation programs, in order to minimise the risks of falls and to prevent their adverse health consequences, such as fractures, and the resulting effects of functional limitations occurring in all organs and systems of the human body. Notably, at present, post-COVID-19 therapy largely focuses on respiratory rehabilitation, breathing exercises and general fitness exercise ^41–45^, whereas balance and postural training seem to be overlooked. However, our findings show that there is a need for post-COVID-19 rehabilitation programs to address this problem and focus on balance training to a greater extent. The assessment showed greater balance impairments in the trials with eyes closed, which suggests that in designing rehabilitation programs for those recovered from COVID-19 infections it is necessary to pay more attention to balance training with no visual control. What is more, our findings also suggest that in designing exercise with double-leg support and single-leg support it is necessary to particularly focus on lateral coordinates and on anterior–posterior coordinates, respectively. These recommendations may be useful for clinicians, doctors and physiotherapists designing interventions for COVID-19 survivors. Furthermore, our study has shown that young adults present with balance impairment after recovery from mild COVID-19. If these findings are reasonably generalised to other populations, it is likely that COVID-19 of any severity may produce the same or more serious effects in older age groups, also significantly increasing the risk of falls in older people. Therefore, it is necessary to continue the research taking into account elderly subjects, since the risk of falls and the resulting fractures is particularly high in this population. Consequently, further research is needed to investigate this issue in more detail, in order to facilitate more accurate diagnoses of postural stability problems and to enable more effective rehabilitation of affected patients.

### Limitations

This study presents certain limitations. Firstly, it involved a narrow age cohort of healthy young adults (age 19–26 years) who had not needed hospitalisation as they had experienced mild symptoms of COVID-19, including fatigue, persisting on average for almost three weeks. Therefore, it is necessary to continue the related research taking into account more age groups, including older individuals, children and adolescents, as well as individuals who suffered from a more severe course of the disease or were hospitalised due to COVID-19. Secondly, the mean time from infection in our study group was just over 4.5 months. It would be worthwhile to conduct a comparative study involving groups of patients at an early stage and over 6 months after the illness. Furthermore, the subjects were assessed only once but the results that we acquired suggest a need for a follow-up study investigating long-term effects of COVID-19 on postural stability; the findings also show that it is necessary to accurately determine the causes leading to problems with balance.

## Conclusions

The current study, conducted in south-eastern Poland, shows that mild COVID-19 may lead to balance impairments in young adults. The findings show statistically significant differences in postural stability between individuals who recovered from mild COVID-19 and healthy controls. The results of this study are important because they show a need for and can be used in developing balance rehabilitation programs in order to minimise risks of falls, and may be helpful in preventing their adverse health-related effects. Further research should take into account more age groups, and patients who experienced severe COVID-19, and should investigate long-term consequences of COVID-19 manifesting as balance problems.

## Data Availability

The datasets used and/or analysed during the current study available from the corresponding author on reasonable request.
